# Longevity of Atlantic Sharpnose Sharks *Rhizoprionodon terraenovae* and Blacknose Sharks *Carcharhinus acronotus* in the western North Atlantic Ocean based on tag-recapture data and direct age estimates

**DOI:** 10.12688/f1000research.4767.2

**Published:** 2015-01-27

**Authors:** Bryan S. Frazier, William B. Driggers III, Glenn F. Ulrich

**Affiliations:** 1 South Carolina Department of Natural Resources, Marine Resources Research Institute, Charleston, SC, 29412, USA; 2 National Marine Fisheries Service, Southeast Fisheries Science Center, Mississippi Laboratories, Pascagoula, MS, 39567, USA

**Keywords:** Rhizoprionodon, Carcharhinus, shark, longevity

## Abstract

Longevity of *Rhizoprionodon terraenovae* and *Carcharhinus acronotus* in the western North Atlantic Ocean was examined using direct age estimates from vertebral sections and tag-recapture data. Time-at-liberty ranged from 7.7-14.0 years (mean =10.1) for *R. terraenovae* and 10.9-12.8 years (mean =11.9) for *C. acronotus*. Maximum estimated longevity was determined to be 19.8 years through tag-recapture data and 18.5 years from direct age estimates for *R. terraenovae* and 22.8 years through tag-recapture data and 20.5 years through direct age estimates for *C. acronotus*. These longevity estimates represent a large increase over previous estimates and may have significant effects on analyses that depend on longevity including lifetime fecundity, mortality rates, demographic analyses and stock assessments.

## Introduction

Tag-recapture data grant researchers the opportunity to synthesize a vast array of information for species they are studying. Sharks, in particular, are well suited for tagging studies due to their migratory behavior, longevity, and relatively large size, which allows tagging at all life stages ( Bonham *et al.*, 1949). Valuable information gained from recapture data includes greater understanding of species-specific migratory patterns, stock structure, spatial and temporal distribution, site fidelity/residence times, and life histories ( Kohler & Turner, 2001).

Among life history parameters needed to best manage populations of fishes, robust longevity estimates are of paramount importance, particularly for iteroparous species, such as sharks. For example, assuming age at maturity, reproductive periodicity, and brood size are constant, the lifetime fecundity of a given species is directly linked to its lifespan. Longevity estimates for sharks are generally derived from von Bertalanffy Growth Function (VBGF) parameter estimates (i.e. growth constant); however, VBGF parameter estimates can be heavily affected by low sample sizes, incomplete sampling among size classes and/or difficulties associated with age estimation of large individuals ( Goldman, 2004; Francis *et al.*, 2007). Obtaining accurate longevity estimates can be hampered by the low probability of catching older individuals as they represent a small portion of the entire population, difficulty associated with capturing large specimens that are more capable of escaping gear than smaller conspecifics, and the reduction of older individuals in the population due to fishing pressure ( Bishop *et al.*, 2006). Additionally, age underestimation has been documented in multiple shark species including long-lived species such as the Porbeagle, *Lamna nasus* (Bonnaterre, 1788) ( Francis *et al.*, 2007) and the Australian School Shark, *Galeorhinus galeus* (L. 1758) ( Kalish & Johnston, 2001), and species with intermediate life histories such as the Bonnethead, *Sphyrna tiburo* (L. 1758) ( Frazier *et al.*, 2014). Due to the above factors, tag-recapture records, when available, are the most reliable source of longevity data as maximum time-at-liberty for any individual within the population would represent the highest directly known longevity. Based on tag-recapture data and direct age estimates, herein we report on the longevity of Atlantic Sharpnose Sharks *Rhizoprionodon terraenovae* (Richardson, 1836) and Blacknose Sharks *Carcharhinus acronotus* (Poey, 1860), both of which are common in the coastal waters off the southeastern United States.

## Methods

Sharks were captured and tagged during survey operations conducted by the South Carolina Department of Natural Resources Adult Red Drum and Coastal Shark Longline Program (SCDNR) (see Ulrich *et al.*, 2007 for longline protocol). Collection, handling and tagging of specimens was authorized and controlled under the SCDNR scientific permit issued to employees of SCDNR.

Upon capture, the fork length (FL) and sex of each shark was recorded. For those sharks deemed to be in good health, a Hallprint ^©^ nylon dart tag, labeled with unique identifying numbers, was inserted into the dorsal musculature at the base of the first dorsal fin prior to release. As tagged sharks were at liberty in the wild, recaptured specimens were reported by a number of sources, including recreational anglers, commercial fishermen and SCDNR. Estimated measurements at recapture were obtained from recreational and commercial fishermen and direct measurements were recorded from specimens recaptured by SCDNR. When possible, a sample of 8–10 vertebrae were removed from the cervical region of the vertebral column from recaptured sharks.

In the case of recaptured specimens from commercial fishermen, sharks were already deceased and vertebrae were removed. In the case of recaptured specimens from SCDNR, a IACUC protocol approved for graduate students who had previously worked with SCDNR on elasmobranch studies was followed; the vertebral column was severed by serrated knife in two cervical locations.

To estimate age at recapture, vertebrae were prepared for analyses following the protocol of Frazier *et al.*, 2014. Initial vertebral growth band counts were conducted by three unbiased readers with no knowledge of specimen length or time-at-liberty. If band counts differed among readers, sections were independently re-read until agreement was reached. Age at recapture was estimated under the assumptions: (1) the birthmark was formed prior or shortly after parturition, (2) the second band was formed 6 months later during the first winter, and (3) the third band was formed 1 year later. Therefore, we subtracted 1.5 from each total band count to calculate age in years (e.g. Loefer & Sedberry, 2003; Driggers *et al.*, 2004).

Age estimates at recapture were also determined by adding time-at-liberty to backtransformed ages at tagging. Species and sex-specific von Bertalanffy growth function parameters from Loefer & Sedberry, 2003 ( *R. terraenovae*) and Driggers *et al.*, 2004 ( *C. acronotus*) were used to backtransform age at tagging using the following equation:

$$\text{Age=}\left(\frac{\text{1n}\left(\text{1-}\frac{L_{t}}{L_{\infty }}\right)}{\text{-k}}\right){\text{+t}}_{\text{o}}$$

    Where:

*L _t_* = length at age t,

*L _∞_* = theoretical maximum length,

*k* = coefficient of growth,

*t _o_* = theoretical age at which length equals zero.

A paired t-test was used to determine if age estimates from vertebral sections were significantly different than backtransformed age estimates. Statistical results were considered significant at α < 0.05.

## Results

### *Rhizoprionodon terraenovae*

**Figure 1.**
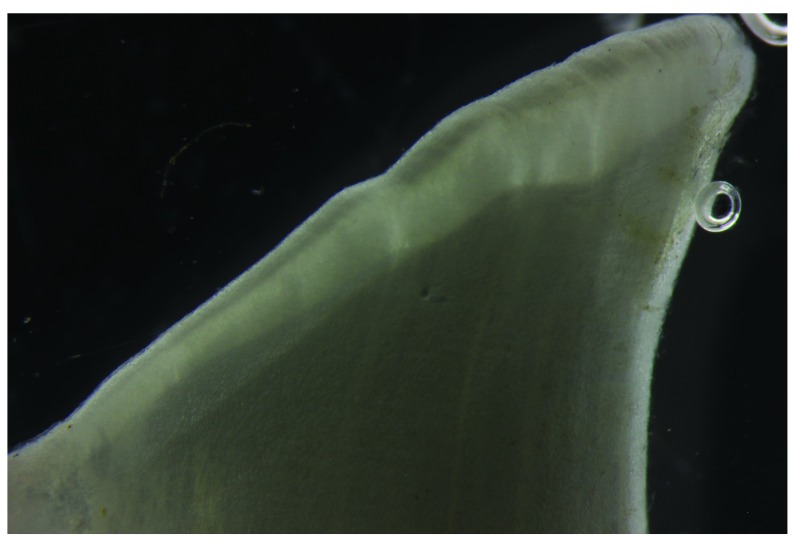
Sectioned vertebra from Atlantic Sharpnose Shark *Rhizoprionodon terraenovae* L5242. Consensus band count was 20 band pairs (age = 18.5 years).

From 1993 to 1998 a total of 3,419 *R. terraenovae* were tagged and released, of these 155 were recaptured. Five *R. terraenovae* (four male and one female) were recaptured with times at liberty ranging from 7.7 to 14.0 years (mean ± S.D. = 10.1 ± 2.7). Length at initial tagging, and when available, length at recapture are listed in Table 1. Backtransformed age at tagging and recapture ranged from 3.8–10.3 years and from 11.8 to 19.8 years, respectively ( Table 1). Vertebrae were sampled from Shark L5242 and the direct age estimate from the vertebral section was 18.5 years old ( Figure 1). Precise measurements were taken for L5242 at tagging and recapture; over 12.1 years-at-liberty this shark grew 28 mm (2.3 mm/year). All five sharks were recaptured within 15 km of initial tagging.

**Table 1.** Initial tagging and recapture information for Atlantic Sharpnose Sharks *Rhizoprionodon terraenovae* and Blacknose Sharks *Carcharhinus acronotus* long-term recaptures from the South Carolina Department of Natural Resources Adult Red Drum and Coastal Shark Longline Program. Fork length (FL) at initial tagging, length at recapture, growth, days and years at liberty, backtransformed age at initial tagging and recapture and direct age estimates are provided.

Species	Tag #	Initial FL (mm)	Recapture FL (mm)	Growth (mm)	Days at Liberty	Years at Liberty	Sex	Backtransformed Age at Initial Tagging	Backtransformed Age at Recapture	Direct Age Estimate
*R. terraenovae*	L2881	776	828*	52	5098	14.0	Male	5.2	19.2	-
*R. terraenovae*	L4774	815	883*	68	3237	8.9	Female	10.1	19.0	-
*R. terraenovae*	L5173	737	-	-	2937	8.0	Male	3.8	11.8	-
*R. terraenovae*	L5242	802	830	28	4438	12.1	Male	7.7	19.8	18.5
*R. terraenovae*	L7250	810	838*	28	2806	7.7	Male	10.3	18.0	-
*C. acronotus*	L2910	876	960	84	4352	11.9	Male	4.5	16.4	15.5
*C. acronotus*	L1515	878	1055	177	4678	12.8	Female	4.2	17.0	14.5
*C. acronotus*	L3384	1020	-	-	3986	10.9	Male	11.9	22.8	20.5

*Denotes approximate measurements as provided by recreational anglers.

### *Carcharhinus acronotus*

**Figure 2.**
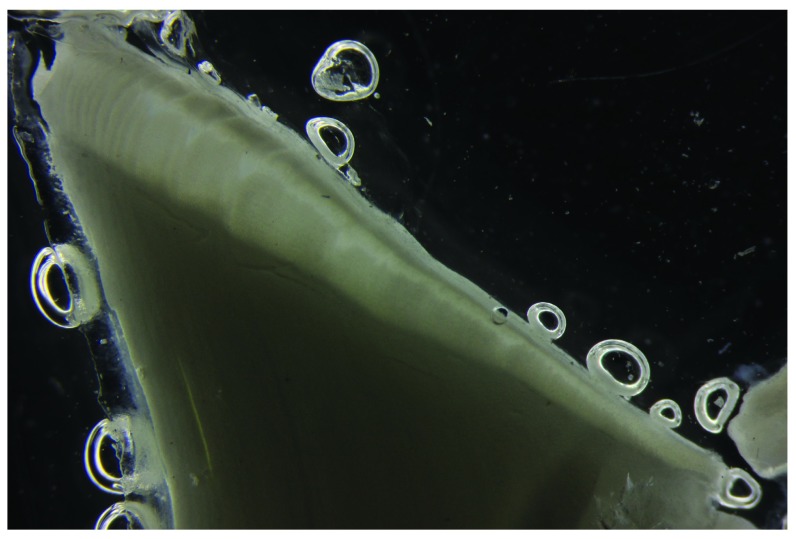
Sectioned vertebra from Blacknose Shark *Carcharhinus acronotus* L3384. Consensus band count was 22 band pairs (age = 20.5 years).

From 1993 to 2013 a total of 1,537 *C. acronotus* were tagged and released, of these 24 were recaptured. Three *C. acronotus* were recaptured (two males and one female) with times at liberty ranging from 10.9 to 12.8 years (mean ± S.D. = 11.9 ± 1.0). Backtransformed age at tagging and recapture ranged from 4.2 to 11.9 years and 16.4 to 22.8 years, respectively ( Table 1). Vertebrae were obtained from all recaptured *C. acronotus*. Age estimates from vertebral sections ranged from 14.5 to 20.5 years ( Figure 2). Precise measurements were taken at capture and recapture for sharks L2910 and L1515. L2910 was at liberty for 12.0 years and grew 84 mm (7.0 mm/year). L1515 was at liberty for 12.8 years and grew 177 mm (13.8 mm/year). All recaptured *C. acronotus* were recovered within 15 km of initial tagging.

### Age estimates and comparisons

In recaptures with both direct and backtransformed age estimates, backtransformed age estimates were significantly larger (paired t-test, *t* = 4.82, *P* = 0.02) ( Table 1). From direct age estimates, *R. terraenovae* observed maximum longevity increased by 9.5 years for males (previously 9+ years [ Loefer & Sedberry, 2003]), and no vertebrae were available from female recaptures ( Table 2).

**Table 2.** Observed maximum longevity from sectioned age estimates, and backtransformed maximum longevity from tag and recapture data for Atlantic Sharpnose Sharks *Rhizoprionodon terraenovae* and Blacknose Sharks *Carcharhinus acronotus*.

Species	Study	Sex	Observed Maximum Longevity (years)	Backtransformed Maximum Longevity (years)
*R. terraenovae*	western North Atlantic Loefer & Sedberry, 2003	Female	11+	-
		Male	9+	-
*R. terraenovae*	Current	Female	-	22.9
		Male	18.0	19.8
*C. acronotus*	western North Atlantic Driggers *et al.*, 2004	Female	12.5	-
		Male	10.5	-
*C. acronotus*	Current	Female	14.5	17.0
		Male	20.5	22.8

*C. acronotus* observed maximum longevity from sectioned age estimates increased by 2 years for females and 8 years for males (12.5 and 10.5 years for females and males respectively [Driggers *et al.*, 2001]). Backtransformed maximum longevity was 4.5 years older than published estimates for females and 12.3 years older for males.

## Discussion

The backtransformed and direct age estimates documented herein greatly increase the known longevity for *R. terraenovae* and *C. acronotus*, which could significantly affect population dynamics models that include longevity as a parameter. Interestingly, the maximum directly estimated ages for both species were associated with male sharks. A review of published shark age and growth studies shows that, in most studies, females have a greater or equal longevity than males (e.g. Carlson & Baremore, 2003; Carlson & Parsons, 1997; Carlson *et al.*, 1999; Driggers *et al.*, 2004; Drymon *et al.*, 2006; Frazier *et al.*, 2014). Therefore, we believe that we did not sample older females and suggest that the longevity estimates we observed for each species be applied to males and females.

Backtransformed age estimates from recaptures were in all cases larger than direct age estimates. This could be evidence that direct ages were underestimated, however direct age estimates only differed by an average of 1.8 years (range 1.0–2.5 years). Observed differences could be due to low sample size or individual variability in growth. The rearranged VBGF gives an estimate of average age-at-length based on parameter estimates. Therefore, specimens could have been younger or older than the average estimated age at time of tagging thus accounting for the discrepancy. Conversely, if the species-specific VBGF do not adequately describe the growth of *R. terraenovae* and *C. acronotus* then the observed differences would be expected. However, the growth models utilized were from studies with robust sample sizes that included all size classes ( Loefer & Sedberry, 2003; Driggers *et al.*, 2004).

The age estimates from this study greatly increase longevity for both species; however, actual life spans could be even longer. The specimens recaptured from this study were tagged in the first few years of the SCDNR longline program (1994–1996). Given project species-specific recapture rates of less than 3% and reported tag shedding rates for nylon dart tags as high as 41% to 63% ( Xiao *et al.*, 1999), the chances of recapturing a shark at liberty for 10+ years are small. The fact that eight were encountered and all exceeded published longevity estimates lends support to this assertion. The data gathered from these recaptures also highlights the importance of continuing long term surveys and tagging efforts. While return rates of tagged sharks are notoriously low, our data demonstrate that continued tagging efforts are essential to provide the most up to date and reliable estimates of maximum longevity. Ideally, to obtain the best possible longevity estimates for a given species, future efforts should focus on tagging neonate sharks with the hope of recapturing them as they approach the end of their lifespans.
