# Supplementary material for: Longevity of Atlantic Sharpnose Sharks Rhizoprionodon terraenovae and Blacknose Sharks Carcharhinus acronotus in the western North Atlantic Ocean based on tag-recapture data and direct age estimates
Source: F1000Res. 2015 Jan 27;3:190. Originally published 2014 Aug 12. [Version 2] doi: 10.12688/f1000research.4767.2 (PMC4314659; doi:10.12688/f1000research.4767.2)
Supplement: Peer review history

# Peer review: Referee response for version 2

**DOI:** 10.5256/f1000research.5743.r7492

**Published:** 2015-01-29

**Authors:** Alastair Harry

**Affiliations:** 1 Centre for Sustainable Tropical Fisheries and Aquaculture, James Cook University, Townsville, Australia

I think the changes made have strengthened the manuscript and have no further comments.

I have read this submission. I believe that I have an appropriate level of expertise to confirm that it is of an acceptable scientific standard.

# Peer review: Referee response for version 1

**DOI:** 10.5256/f1000research.5090.r5801

**Published:** 2014-09-02

**Authors:** Marcus Drymon

**Affiliations:** 1 Department of Marine Sciences, University of South Alabama, Mobile, AL, USA

This manuscript reports increased longevity for two common species of small coastal sharks (Atlantic Sharpnose Shark *Rhizoprionodon terraenovae* and Blacknose Shark *Carcharhinus acronotus*) based on tag recapture data. These findings are particularly interesting given the previously assumed longevity of these species, and are clearly relevant to population models that include longevity as a parameter for these species. This note is clearly written, and conclusions from this work are appropriately presented; moreover, these data illustrate the benefit of long-term fisheries-independent monitoring and tagging programs for long-lived species such as sharks.

These data are clear, concise, and important to the continued successful management of these two species; as such, they are worthy of publication. The two tables presented are sufficient to convey the data discussed. Given the straightforward nature of these data and their appropriate interpretation by the Authors, I have no major comments. There are two typos in the first paragraph of the Discussion (detailed below) which should be addressed.    

1. In the sentence “A review of published shark age and growth studies shows that, in most studies, ***that*** females have a greater…,” remove ***that.***
2. In the sentence “Therefore, we believe that we did not sample older females and suggest that the ***longevity estimates maximum observed longevity*** we observed for each species…,” revise “ ***longevity estimates maximum observed longevity” ***as desired.

I have read this submission. I believe that I have an appropriate level of expertise to confirm that it is of an acceptable scientific standard.

# Response

**Published:** 2015-01-21

**Authors:** Bryan Frazier

**Affiliations:** South Carolina Department of Natural Resources, USA

The authors thank the reviewer for the suggested edits, all have been incorporated.

# Peer review: Referee response for version 1

**DOI:** 10.5256/f1000research.5090.r5802

**Published:** 2014-08-26

**Authors:** Alastair Harry

**Affiliations:** 1 Centre for Sustainable Tropical Fisheries and Aquaculture, James Cook University, Townsville, Australia

This research note presents data on the long-term recapture of seven individuals of two species of coastal sharks from the Northwest Atlantic; *Rhizoprionodon terranovae*, and the *Carcharhinus acronotus*. Based on their length at tagging, all individuals appeared to be either mature or close to maturity, but were recaptured between 7.7 and 12.8 years later. As neither species was reported to be particularly long-lived these results are interesting, and provide evidence that both species live longer than currently thought.  Age was also estimated and compared in four of the samples by reading sectioned vertebrae. These data contribute to growing evidence that longevity of sharks is often underestimated when based on vertebral ageing methods, and that some aspects of elasmobranch demography that have been inferred from these studies (e.g. survival) may be biased. I’m glad the authors have taken the time and effort to document these observations, as there are relatively few long-terms studies of this kind.

While I think the data themselves are worthy of publication, I don’t think any of the subsequent analysis contributed hugely to the paper. Published growth curves are used to estimate the age at tagging, which is then added to the time at liberty to get a ‘backtransformed age at recapture’. Unfortunately, neither of these original growth studies presented the model variance, a detailed description of the data, or even any plots of the size at age data! Without this information, it is hard to get an idea of how variable size-at-age is for these species (and it is clearly quite variable), or the representativeness of the original sampling, etc.  Personally, I think other information could be more useful, like photographs of the two oldest vertebrae sections for reference.

I recommend two revisions to the research note. Firstly, some more background information is required on the tagging study itself. It is not stated how many sharks were tagged and recaptured over the duration of the whole study, nor is it clear why these seven were chosen specifically (presumably they weren't the only recaptures). Secondly, I disagree that Fabens’ theoretical method for estimating longevity is the usual method of estimating longevity. I don’t see the value with making comparisons to theoretical longevity later on in the paper and find this confusing. For example, the ‘theoretical maximum longevities’ of *R. terranovae *are stated to be 7.1 and 6.9 years for females and males respectively, but Loefer and Sedberry (2003) reported actual longevities of 10 and 9 years.

I have read this submission. I believe that I have an appropriate level of expertise to confirm that it is of an acceptable scientific standard, however I have significant reservations, as outlined above.

# Response

**Published:** 2015-01-27

**Authors:** Bryan Frazier

**Affiliations:** South Carolina Department of Natural Resources, USA

The authors appreciate the suggested revisions offered by the reviewer. We agree that it is unfortunate that the original age and growth studies did not present individual length at age data with their published growth curves. While we felt it most appropriate to cite the peer reviewed publications, it should be noted that these data are available for *Carcharhinus acronotus* (SEDAR 21 DW-36, http://www.sefsc.noaa.gov/sedar/download/S21_DW_36.pdf?id=DOCUMENT) as a working paper for United States SouthEast Data, Assessment and Review *C. acronotus* stock assessment. Unfortunately, there are no publicly accessible documents showing variation in length-at-age for *Rhizoprionodon terraenovae*. The authors agree with the reviewer that images of the oldest aged vertebra for each species are appropriate, and we have added those figures. 

As requested, the authors have added minimal background information detailing the total tagged and recaptured of each species. The authors feel adding additional background information beyond this would not be of value to readers. 

The recaptures that were used for this note were chosen due to their lengthy times-at-liberty. Recaptures with shorter times-at-liberty were not used as they would be less informative given variability in length-at-age. 

The authors agree with the reviewer that including Faben's theoretical maximum longevities were confusing and we have removed these data from the note.
